# Cardiac metastasis mimicking STEMI—impact of point-of-care ultrasound on clinical decision-making: A case report

**DOI:** 10.3389/fcvm.2023.1098154

**Published:** 2023-03-22

**Authors:** Anh Ngoc Le, Anh Van Nguyen, Trang Ngoc Nguyen, James N. Kirkpatrick, Huyen Thi Nguyen, Hoai Thi Thu Nguyen

**Affiliations:** ^1^ Vietnam National Heart Institute, Bach Mai Hospital, Hanoi, Vietnam; ^2^ Department of Cardiology, Hanoi Medical University, Hanoi, Vietnam; ^3^ Radiology Center, Bach Mai Hospital, Hanoi, Vietnam; ^4^ Cardiovascular Division, Department of Medicine, University of Washington Medical Center, Seattle, WA, United States; ^5^ Department of Bioethics and Humanities, University of Washington Medical Center, Seattle, WA, United States; ^6^ Department of Internal Medicine, VNU – University of Medicine and Pharmacy, Hanoi, Vietnam

**Keywords:** cardiac metastasis, ST-segmental elevation, point-of-care ultrasound, myocardial infarction, case report

## Abstract

**Introduction:**

The manifestations of cardiac metastases are extremely variable depending on their location and extension.

**Case presentation:**

A 62-year-old man was admitted to the cardiac emergency department presenting with chest pain, worsening shortness of breath and palpitations. He had a history of esophageal squamous cell carcinoma treated with chemoradiotherapy, and he was not diagnosed with cardiovascular disease before. The electrocardiogram showed significant ST-segment elevations in leads II, III, and aVF. Initially, the patient was diagnosed with ST-segment elevation myocardial infarction. A cardiac point-of-care ultrasound was performed immediately revealing two large heterogeneous masses in the left ventricular wall and the apex, which changed the diagnosis and the management strategy. There was no significant change in serial cardiac biomarkers in the setting of persistent STE. Thoracic computed tomography and cardiac magnetic resonance confirmed that the patient was suffering from cardiac and lung metastases.

**Conclusion:**

ECG findings of localized and prolonged STE without Q waves or changes in biomarkers may suggest myocardial tumor invasion, especially in the cancer setting. Cardiac point-of-care ultrasound is an effective, convenient, noninvasive imaging modality to guide real-time clinical decision-making.

## Introduction

Cardiac metastases account for 95% to 99% of cardiac tumors (1, 2). The exact incidence of cardiac metastatic disease is unknown. A study of post-mortem examinations showed that the proportion of in-hospital deceased patients who had cardiac metastases was 9.1% which was highly variable depending on the types of primary tumors, from 1.0% in prostate carcinoma to 48.4% in mesothelioma (2). Clinical silence is prevalent in most cases (3). The manifestations of cardiac metastases are extremely variable depending on their location and extension (4, 5). We report a case of myocardial metastatic with ST-segment elevation (STE) on electrocardiogram (ECG) and intramyocardial masses noted on cardiac point-of-care ultrasound (POCUS).

## Case presentation

A 62-year-old man without cardiac history was admitted to the cardiac emergency department complaining of chest pain, worsening shortness of breath, and palpitations for 5–6 weeks. His medical history included esophageal squamous cell carcinoma (ESCC) which was treated with chemoradiotherapy for 1.5 years. The treatment strategy was completed 6 months ago.

**Figure 1:**
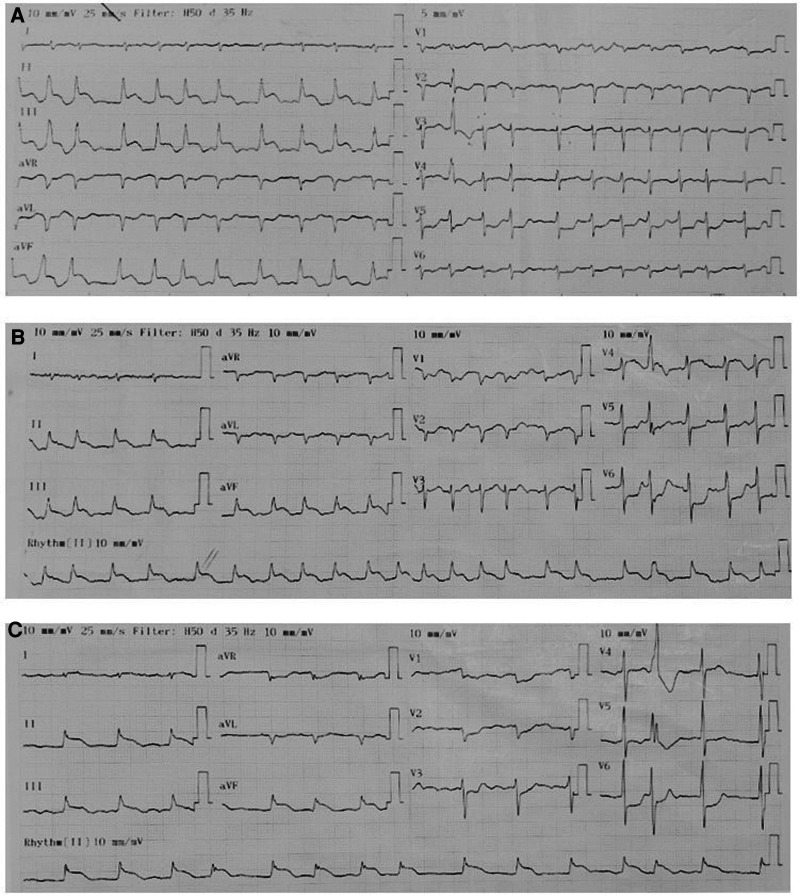
Serial ECGs. The pattern of ECGs was persistent, including atrial fibrillation, right axis deviation, poor R-wave progression, profound STE in leads II, III, and aVF with the presence of reciprocal ST depression in the precordial leads. (**A)** On admission. (**B)** 1 h later. (**C)** Before discharge.

On admission, the physical examination revealed extreme frailty with a BMI of 16.2 kg/m^2^, heart failure signs including elevated jugular venous pressure, rales in the lungs, peripheral edema, hepatomegaly, and low blood pressure of 75/40 mmHg. Oxygen saturation was 89% while breathing ambient air. A 12-lead ECG showed rapid atrial fibrillation, and significant convex STE in leads II, III, and aVF with the presence of reciprocal ST depression in the precordial leads (Figure 1A). No prior ECG was available for comparison. Initially, the patient was diagnosed with ST-segment elevation myocardial infarction (STEMI). However, because he had hemodynamic instability and had a history of ESCC, we decided to perform a cardiac POCUS immediately for a more comprehensive assessment before transferring the patient to the catheterization laboratory for urgent percutaneous coronary intervention (PCI). The echocardiogram showed two large masses in the left ventricular wall and the apex. The left ventricular systolic function was low. Because of these echo findings, the PCI was deferred. Treatment was initiated with oxygen, dobutamine, furosemide, digitalis, and enoxaparin.

**Figure 2:**
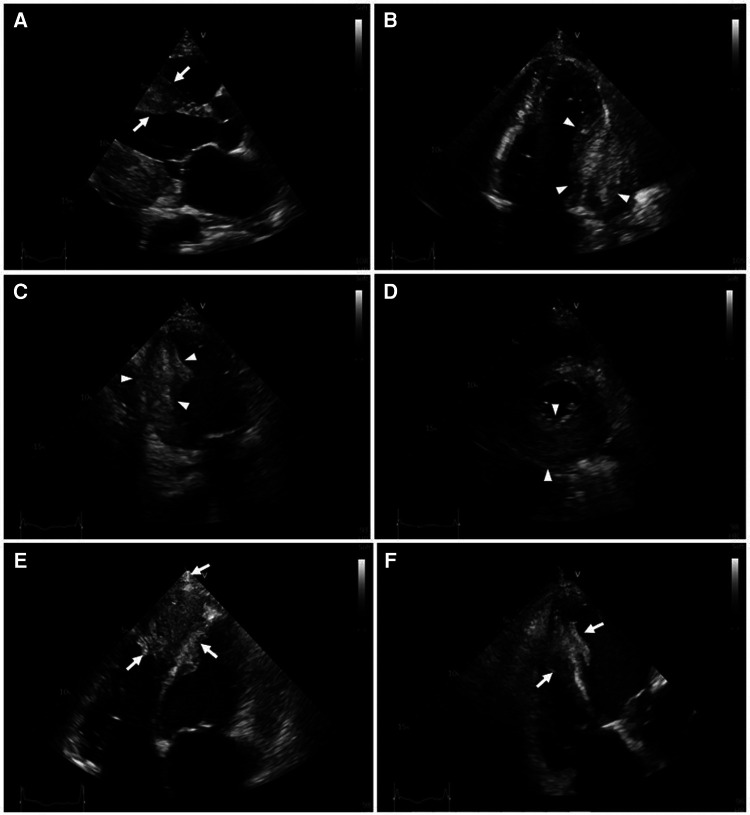
Transthoracic two-dimensional echocardiography (2D-TTE). 2D-TTE reveals two large heterogeneous masses characterized by ill-defined borders: one involving the lateral, inferior and posterior portions of the left ventricular wall *(white arrowheads)*, and another involving the apex and ventricular septum *(white arrows)*. (**A)** Parasternal long-axis view. (**B)** Four-chamber view. (**C)** Two-chamber view. (**D)** Parasternal short-axis view at the papillary muscle level. (**E)** Modified four-chamber view. (**F)** RV-focused apical four-chamber view.

Serial hs-cTnT measurements and ECGs were obtained. A comprehensive transthoracic echocardiogram (TTE) was performed on the following day. On serial ECGs, STE persisted and there was no development of pathological Q waves (Figures 1B, C). Laboratory studies showed that hs-cTnT stabilized at a high level: on admission 0 h 173.1 ng/L, 1 h 167.3 ng/L, 3 h 159 ng/L, on discharge 180 ng/L (<14 ng/L); elevated N-terminal pro-B-type brain natriuretic peptide (NT-pro-BNP) was 1391 pmol/L < 14.47 pmol/L). TTE unveiled two large heterogeneous masses, which were characterized by ill-defined borders: one involved the lateral, inferior and posterior portion of the left ventricular wall, and another involved the apex and ventricular septum portion (Figure 2). The involved ventricular walls were significantly thickened and akinetic, which did not correspond to coronary perfusion territories. Left ventricular systolic dysfunction (biplane left ventricular ejection fraction of 35%), and right ventricular systolic dysfunction (tricuspid annular plane systolic excursion of 15 mm and fractional area change of 30%) were detected.

This patient was referred for a thoracic multidetector computed tomography (MDCT) scan and cardiac magnetic resonance imaging (CMR) for further evaluation of imaging characteristics of the cardiac masses, and other lesions.

**Figure 3:**
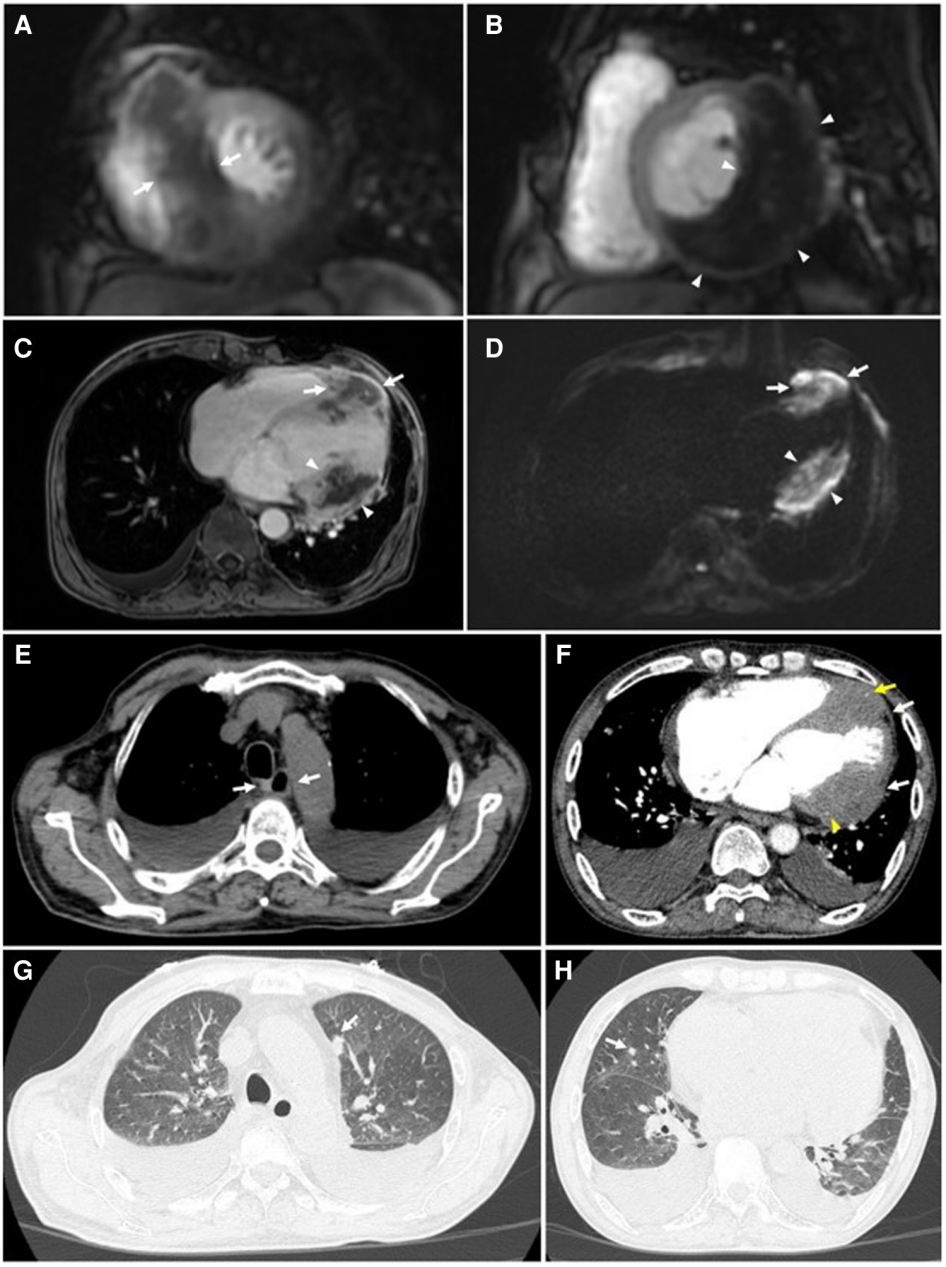
Cardiac magnetic resonance and thoracic computed tomography were obtained 6 months after completion of chemoradiotherapy. CMR: (**A to D)** Tumors of the interventricular septum *(white arrows)* and inferior-lateral left ventricular wall *(white arrowheads)* do not demonstrate enhancement on short-axis first-pass perfusion images (**A & B**) and axial post-contrast T1-weighted images (**C**) suggestive of necrosis. These lesions demonstrate restricted diffusion on the axial diffusion-weighted images (DWI) (**D)**. (**E)** No abnormal thickening of the esophageal wall is seen on the mediastinal window *(white arrows).* (**F)** Tumors of the interventricular septum and inferior-lateral left ventricular wall invade the left anterior descending (LAD) artery *(yellow arrows)* and left circumflex (LCx) artery *(yellow arrowheads)*, respectively. These lesions also extend to the pericardium *(white arrows).* (**G & H)** Metastatic nodules in the upper lobe of the left lung and the middle lobe of the right lung are seen on the lung window *(white arrows)*.

**Figure 4:**
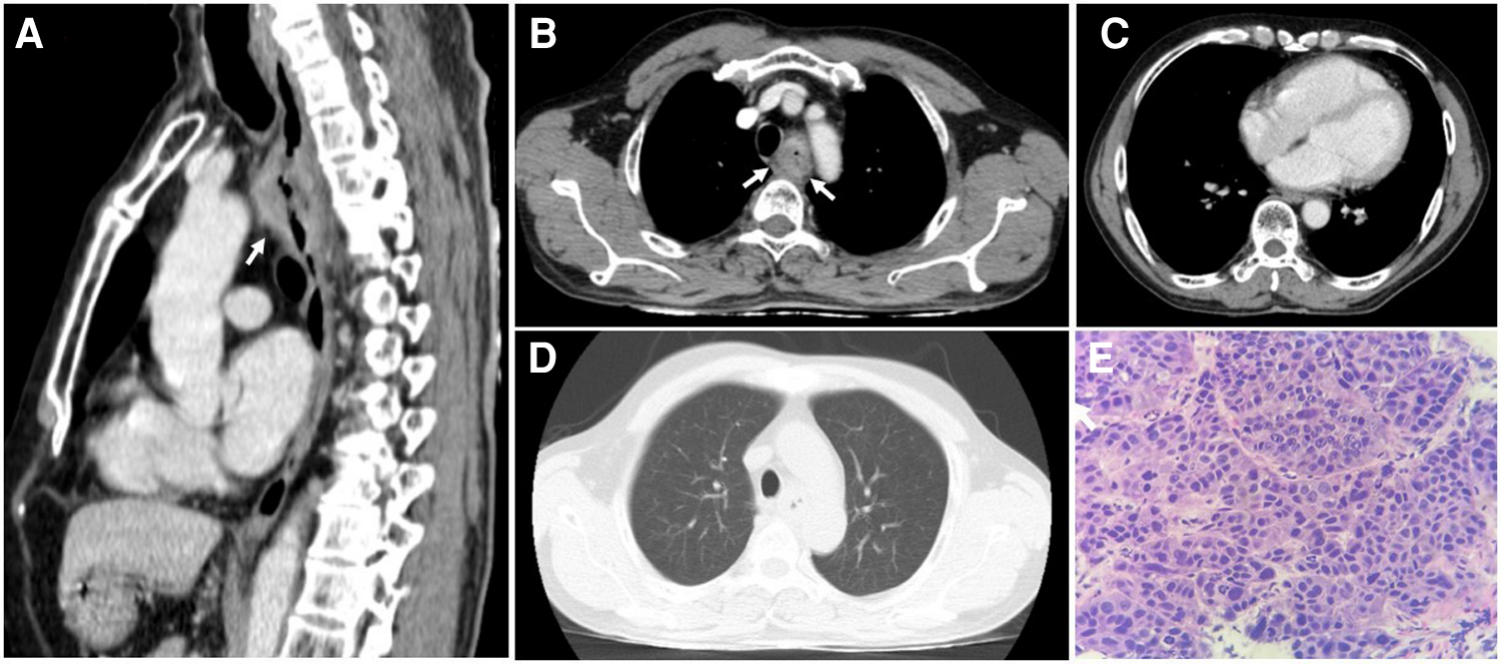
The thoracic computed tomography and histopathology before treatment strategy for esophageal squamous cell carcinoma. (**A & B)** The sagittal and axial planes demonstrate that the upper esophageal tumor does not invade the surrounding structures *(white arrows).* (**C)** The mediastinal window demonstrates normal thickness and density of ventricular and atrial walls. (**D)** No parenchymal lesions are seen on the lung window. (**E)** Hematoxylin and eosin-stained esophageal tissue at 400 × magnification showed tumor cells that possess enlarged, hyperchromasia, coarse nucleus; visible nucleoli and large cytoplasm. The tumor cells demonstrate disarray and loss of polarity in the desmoplastic stroma.

On thoracic MDCT, the mediastinal window showed infiltrative lesions causing abnormal wall thickening of the interventricular septum toward the apex, as well as of the inferior, posterior, and lateral walls of the left ventricle corresponding to the lesions observed on TTE. These lesions extended beyond the myocardium to the pericardial fat. The left anterior descending (LAD) artery and the left circumflex (LCx) artery were encased completely and compressed mildly. The adjacent pericardium was irregularly thickened (Figure 3F). These lesions were new in comparison with the pre-treatment film (Figure 4C). The ESCC was significantly diminished, and there was no longer abnormal esophageal wall thickening (Figures 4A, B; Figure 3E). On lung window, two new well-defined solid nodules in the left upper lobe and the right middle lobe were detected (Figures 3G, H) compared to before treatment (Figure 4D). On CMR, two tumors enhanced mildly on first pass perfusion images but had peripheral heterogeneous enhancement in post-contrast T1-weighted images with central necrosis. In addition, the tumors appeared to have restricted diffusion on the diffusion-weighted imaging (DWI) (Figure 3 A to D).

Although the patient declined invasive coronary angiography and coronary computed tomography angiography, the unremarkable change in serial cardiac biomarkers in the setting of persistent STE strongly argued against STEMI. It was concluded that the patient suffered from cardiac and lung metastases from ESCC. The changes in ECGs were attributed to the patient's cardiac metastases. Given his disease progression and poor prognosis, the patient refused further diagnosis methods during this admission and wished to pursue palliative treatment. The patient was referred to the local hospital for palliative care. He passed away from multiple organ failure one month later.

## Discussion

Cardiac metastases are usually diagnosed on imaging or autopsy incidentally (1, 2). Primary tumor can metastasize to the heart through four pathways, including hematogeneous, lymphatic, or transvenous spread, or direct invasion. Each metastatic pathway has a different target tissues (6). Pericardial involvement is the most common (69.4%), which may result in pericardial effusion and tamponade, followed by epicardial or myocardial metastases, at 34.2% and 31.8%, respectively (4). A mere 5% of cardiac metastasis involves the endocardium (4). The clinical manifestations of myocardial metastasis depend not only on the degree of metastatic infiltration but also on the location (4). Life-threatening complications include arrhythmias (complete atrioventricular block due to disruption of the cardiac conduction system by cardiac metastases, atrial fibrillation, and ventricular fibrillation, among others), and congestive heart failure (4, 5, 7). Rarely, myocardial metastases may result in cardiac rupture, cardiac tamponade, and sudden death (5). Endocardial and intracavitary lesions may occasionally lead to inflow and outflow obstruction of the heart chambers (4, 5). The coronary arteries may be injured by neoplasm-induced coronary embolism, perivascular compression of the coronary arteries, or invasion of the coronary arteries (4, 5).

Regarding ECG changes in cardiac metastasis, the most common abnormalities are ST-T changes (nonspecific ST-T changes, T wave inversion, ST-segment depression, STE) (8). Myocardial metastasis can result in conduction disturbances, atrial arrhythmias, ventricular arrhythmias, which may be hard to control by antiarrhythmic medications (7–10). Low-voltage and electric alternans may indicate a pericardial effusion and tamponade (8).

STEMI is the most common cause of STE. However, STE can be found in a wide range of conditions, including normal variants, left bundle-branch block, acute pericarditis and myocarditis, electrolytes disturbances, the Brugada syndrome,arrhythmogenic right ventricular cardiomyopathy, early repolarization, transient STE after transthoracic cardioversion, and Prinzmetal's angina (11, 12). STE in the setting of cancer can be seen in many situations, for example, myocardial metastasis, tumor emboli within a coronary artery (13), coronary artery invasion or compression by surrounding metastatic lesions (14) or cancer therapy-related cardiovascular toxicity (coronary vasospasm, accelerated atherosclerosis and plaque rupture, and coronary thrombosis and embolism (15–17).

Our patient presented with localized, persistent STE without development of pathological Q waves. Comprehensive assessment of coronary artery injuries in our patient was not possible because he refused to undergo either coronary computed tomography angiography or coronary angiography. Nevertheless, the stable hs-cTnT and ECG pattern strongly suggested the absence of STEMI.

Although STE related to myocardial metastasis is rare, it has been published in several case reports recently (18–23) and in a large series by Lestuzzi (24). Of note, evolutionary changes occurring in STEMI are not seen in patients with cardiac metastases mimicking STEMI (18, 19, 21–23). These ECGs features seem to be a sign of myocardial metastases. In patients with intracardiac, pericardial, or paracardiac neoplastic masses, 86% of patients with STE suffered from myocardial infiltration detected by two-dimensional echocardiography (24). In addition, the localization of STE reflects the location of the cardiac infiltration (18–24).

The mechanism of this phenomenon is the same as that of STE in STEMI. Malignant cardiac infiltration results in a change in the electrical properties of the myocardium, leading to the differences in the action potential between the involved and the normal myocardium. An injury current results, which manifests on the ECG as STE (21, 25).

Withholding reperfusion therapy in a patient suffering from STEMI for time consuming diagnostic testing is inadvisable as it results in a delay in therapy. Routine echocardiography before primary PCI is therefore not recommended. Emergency echocardiography is indicated in patients with cardiac arrest, cardiogenic shock, hemodynamic instability or suspected mechanical complications, and in cases in which the diagnosis of STEMI is uncertain (26). Clinical use of POCUS has been extensively described in medical literature since the early 2000s (27). One study found that POCUS confirmed a suspected clinical diagnosis in 50% of cases and changed the diagnosis in 23%, with a change in treatment plan in 53% (28). With the evolution of handheld ultrasound systems and the increasing evidence supporting in clinical practice, POCUS is considered as an effective and emerging tool for the frontline clinician. Recently, the use of cardiac POCUS has increased significantly and become standard in many emergency departments and critical care settings (29). The advantages of POCUS including low cost, time efficiency, ease of use, and accuracy are the backbone of its wide implementation in making diagnoses, clinical monitoring, and guiding procedures (30). In the literature, the number of cardiac POCUS publication has increased yearly. The proportion of emergency medicine and cardiology authors were 38.5% and 20.8%, respectively, decreased over the last decade, while those of anesthesiology and critical care have increased (31). In our patient cardiac POCUS changed the diagnosis and the management strategy. This case reinforces the importance of POCUS in real-time clinical decisions, especially in patients with complicated presentation.

## Conclusion

Metastases to the myocardium and pericardium may mimic acute STEMI which requires timely diagnosis and emergent coronary revascularization. Investigating STEMI-mimicker in the right clinical setting can be important before committing patients to reperfusion strategies. ECG findings of localized and prolonged STE may suggest myocardial tumor invasion, especially in the cancer setting. Cardiac POCUS is a convenient, noninvasive imaging modality to guide real-time clinical decision-making.

## Data Availability

The raw data supporting the conclusions of this article will be made available by the authors, without undue reservation.
