# Supplementary material for: Cardiac metastasis mimicking STEMI—impact of point-of-care ultrasound on clinical decision-making: A case report
Source: Front Cardiovasc Med. 2023 Mar 22;10:1098154. doi: 10.3389/fcvm.2023.1098154 (PMC10073711; doi:10.3389/fcvm.2023.1098154)
Supplement: Supplementary file 1 [file Table1.docx]

**Timeline of patient clinical course**

| April 2021 | The patient was diagnosed with esophageal squamous cell carcinoma |
| --- | --- |
| May 2021 to April 2022 | The patient was treated with a combination of radiotherapy and chemotherapy |
| Aug 2022 | The patient had chest pain, worsening shortness of breath, and palpitation |
| 7 Sep 2022 | Hosptalization  ECG on admission showed rapid atrial fibrillation, significant STE in leads II, III, and aVF  The cardiac point-of-care ultrasound revealed two large masses in the left ventricular wall and the apex  The PCI was deferred. Treatment was initiated with oxygen, dobutamine, furosemide, digitalis and enoxaparine.  Serial hs-cTnT (on admission, 1h, 3h): hs-cTnT stabilized at a high level  Serial ECGs (on admission, 1h, 6h): the ECG pattern did not change. |
| 8 Sep 2022 | The 2D TTE revealed two large heterogeneous masses, which were characterized by ill-defined echodensity.  On the MDCT, the mediastinal window showed infiltrative lesions causing abnormal wall thickening of the left ventricle wall corresponding to the lesions observed on TTE. The left anterior descending artery and the left circumflex artery are invaded. On the lung window, there were two new well-defined solid nodules.  ECG: the STE persisted. |
| 19 Sep 2022 | On the CMR imaging, two tumors did not enhance on perfusion images first-pass but had peripheral heterogeneous enhancement in post-contrast T1-weighted images with center necrosis |
| 22 Sep 2022 | ECG before discharge: the STE persisted.  hs-cTnT before discharge remained stabilized at a high level  The patient was referred to the local hospital for palliative care |
| 18 Oct 2022 | The patient passed away from multiple organ failure one month later |
